# A Novel Anti-BoNT/A Neutralizing Antibody Possessed Overlapped Epitope with SV2 and Had Prolonged Half-Life In Vivo

**DOI:** 10.3390/toxins17080376

**Published:** 2025-07-29

**Authors:** Shangde Peng, Naijing Hu, Fenghao Peng, Huirong Mu, Zihan Yi, Cong Xing, Liang Zhang, Wen Hu, Xinyi Zhou, Yan Wen, Jiannan Feng, Chunxia Qiao

**Affiliations:** 1 College of Biotechnology, Campus of Jiangsu University of Science and Technology, Zhenjiang 212100, China; pengshangde@163.com (S.P.); muhuirong@just.edu.cn (H.M.); txk0122@163.com (Z.Y.); zhangl_edu@163.com (L.Z.); m15255359075@163.com (W.H.); zxywsx0601@163.com (X.Z.); 2 State Key Laboratory of National Security Sepcially Needed Medicines, Beijing 100039, China; h547214399@163.com (N.H.); pfh1722@163.com (F.P.); zhangjiehai1124@163.com (J.F.); 3 Joint National Laboratory for Antibody Drug Engineering, Henan University, Kaifeng 475004, China; xingcong2022@163.com

**Keywords:** nanobody, FcRn, BoNT/AHC, SV2, half-life

## Abstract

The C-terminus of the BoNT/A heavy chain (BoNT/AHC) mediates binding to its receptor, SV2, a critical step for toxicity. Antibody inhibition of this interaction enhances neuronal survival. We previously identified a functional anti-BoNT/AHC nanobody, HM. To extend its in vivo half-life, we designed and prepared two Fc-optimized nanoparticles, HM-Fc5 and HM-Fc6. Structural modeling (homology/docking) of the HM Fv-AHC complex predicted that HM engages key AHC residues (Tyr^1155^, Phe^1160^, Ile^1161^, Val^1184^, Asn^1188^, Lys^1189^, Glu^1190^), which overlap with the SV2 binding site. This suggests HM’s protective mechanism involves blocking toxin-receptor binding and cellular entry. HM-Fc5 and HM-Fc6 retained the stability and function of the parental HM antibody while exhibiting prolonged in vivo half-life. These optimized nanobodies offer economical candidates potentially enabling longer dosing intervals, beneficial for prophylaxis or chronic disease treatment. Significance Statement: The purpose of the study is to design and prepare two Fc optimized nanoparticles, HM-Fc5 and HM-Fc6, and predict the key residues involved in the interaction between HMs and AHC. The experimental results showed that HM-Fc5 and HM-Fc6 have the same stability as the parent HM antibody but have a longer half-life in vivo. The key residues Tyr^1155^, Phe^1160^, Ile^1161^, Val^1184^, Asn^1188^, Lys^1189^, and Glu^1190^ overlap with the SV2 binding site. Our experimental results indicate that these nanobody candidates are not only more economical and convenient, but may also have longer dosing intervals, providing strong evidence and reference for prolonging the in vivo half-life of nanomaterials.

## 1. Introduction

Botulinum toxin is a potent neurotoxin produced by *Clostridium botulinum*, which is the most toxic biological toxin known at present [1,2]. Botulinum toxin belongs to metalloproteinase that mainly contains seven serotypes: BoNT/A-G. BoNTs comprise two primary elements: the heavy chain (HC) that binds with receptors at the neuromuscular junction, thereby facilitating the transfer of botulinum toxin into nerve cells, and the light chain (LC), which specifically cleaves SNARE (soluble N-ethylmaleimide-sensitive factor attachment protein receptor) proteins, which are involved in the fusion between vesicles and the cell membrane [3]. The coordinated action of these two components blocks the signal transmission between nerves and muscles, resulting in muscle paralysis [4]. In medicine, botulinum toxin is used to treat excessive muscle tension and some movement disorders, such as torticollis and blepharospasm. In addition, it is widely used in the cosmetic field to reduce wrinkles and facial expression lines [3,5].

Foodborne contamination, [6] wound infection, [7] intestinal poisoning [8,9], and iatrogenic poisoning [7] cause botulinum toxin poisoning in low doses. There have been continuous reports of botulinum toxin poisoning worldwide, with type A botulinum toxin as the main most responsible toxin. Due to its easy preparation, high toxicity, and ease of production and transportation, BoNT has been listed as a category A biological warfare agent by governments and international organizations (CDC, BTWC, FDA, etc.), and is considered as a biological weapon in biological attacks [10]. BoNT/A is now commonly used in the treatment of various muscle spasmodic diseases, such as cerebral palsy, migraine, cervical dystonia, etc. Common side effects include pain, swelling, and bruising at the injection site, as well as the possibility of triggering an inflammatory response in some cases, and new research has shown that the use of antibodies to block BoNT/A has shown strong therapeutic effects in mouse models [11]. 

At present, the primary treatments for botulinum toxin poisoning involve the equine antitoxin serum and immunoglobulin. However, these two antitoxins have some disadvantages such as low yield, instability between batches, and significant side effects. Antibody drugs have a safety profile, efficacy, and minimal side effects. Up until now, thousands of antibody drugs have progressed through clinical trials, with the FDA grant in approval to over 170 antibody drugs for a variety of diseases, including cancer, autoimmune diseases, and infections. Monoclonal antibodies are excellent candidate drugs due to their high safety, weak immunogenicity, and good in vivo tolerance, with numerous research teams announcing their development of potential candidate antibodies [12,13].

Similar to traditional antibodies, nanobodies (Nbs) have also received significant attention in the development of anti-botulinum toxin drugs [14,15]. Nanobodies are variable regions of heavy chain antibodies (HcAbs), also known as single-domain antibodies (sdAbs) or heavy chain variable region domains (VHHs), which are the smallest units with antigen-binding activity. Nanobodies are naturally derived from special animals like camels or sharks. Nanoantibodies have a small molecular weight (about 12–15 kDa) with the advantages of easy expression, good stability, and strong tissue penetration, etc. [16,17].

The atomic-resolution structures of antigen-antibody complexes offer critical insights into their functional mechanisms. This structural precision enables residue-level interrogation of interaction interfaces, thereby elucidating how specific modifications to parental antibody residues enhance affinity. Such mechanistic understanding facilitates the rational engineering of high-efficacy antibody variants [18]. With the development of bioinformatics, based on the features like flexibility, solvent accessibility [19], and amino acid propensity scales [20], many methods have been used for identifying the key interacting residues of antibodies. For example, BIOSYNTH uses peptide technology for epitope analysis, which mimics the native conformation of the target protein by creating a three-dimensional structure of the CLIPS™ peptide structure, thereby identifying the binding site of the target antibody with its target. In addition, studies based on the AlphaFold algorithm show that the algorithm can accurately predict the three-dimensional structure of antibodies, providing new possibilities for antibody characterization and epitope prediction [21].

The study of antibody epitopes is also critical to understanding and responding to emerging pathogens. For example, for the SARS-CoV-2 virus, the researchers analyzed the structural data of the Spike protein antigen-antibody complex and explored the spatial recognition relationship between epitopes and antibodies. Through the analysis of the structural data of 718 pairs of Spike protein antigen-antibody complexes, the researchers found that 94.02% of the epitope loci were distributed in the receptor binding domain (RBD), and the antibody gene fragments were revealed to be prominent in IGHV3-30/IGHJ4 and IGHV1-58/IGHJ3 fragments using bias analysis [22].

The Fc region, a crucial segment of the antibody molecule, binds with the Fc receptor on the cell surface. Mutations within this region can significantly affect the serum half-life and biological activity of the antibody. By optimizing the pH-dependent IgG Fc-FcRn interaction, the researchers designed a variety of Fc variants. Scientists from the Biomedical Research Center of Anam Hospital, Korea University, reported a novel Fc variant, PFc29. At the same time, they identified two mutations in it (Q311R/M428L), which were able to improve pH-dependent binding with FcRn and prolong the persistence of IgG antibodies and Fc fusion proteins. It is expected to be an excellent tool to enhance the pharmacokinetic profile and potency of various therapeutic antibodies and Fc fusion proteins [23]. At present, the strategies to prolong the half-life of IgG mainly focus on Fc region modification, isoelectric point (pI) modification, glycosylation modification, antigen-binding site pH-dependent modification, and antigen-binding calcium ion-dependent modification. [24] The implementation of these strategies has made significant progress in improving the efficacy and safety of therapeutic antibodies and has important implications in reducing the frequency of dosing, reducing the cost of treatment, and improving patient compliance.

Previously, we have selected a type of anti BoNT/A nanobody called HM [25,26] (Chinese patent: ZL202311169052.7) screened from immune camels in the early stage of the laboratory, and determined its high affinity for binding antigens and in vivo neutralizing activity in a mouse model. Based on the crystal structure of BoNT/AHC and the computational model of HM, the interaction mode of BoNT/AHC was theoretically analyzed, a series of BoNT/AHC mutants (AHC1~5) were predicted and designed, and key residues in BoNT/AHC identified by HM were tested. HM is expressed as a VHH-Fc fusion protein (HM-Fc) that has been functionally validated to assess its efficacy and specificity. At the same time, based on the key site between Fc and FcRn, mutants of Fc were designed. HM-Fc mutants Fc5 and Fc6 improve functional outcomes such as half-life, stability, and in vivo biological activity. The results showed that the mutants Fc5 and Fc6 had a longer half-life than HM-FC and maintained the activity of neutralizing botulinum toxin in vivo.

## 2. Results

### 2.1. Prediction of Key Epitopes of Toxin Based on BoNT/A–HM Complex Structure

**Figure 1:**
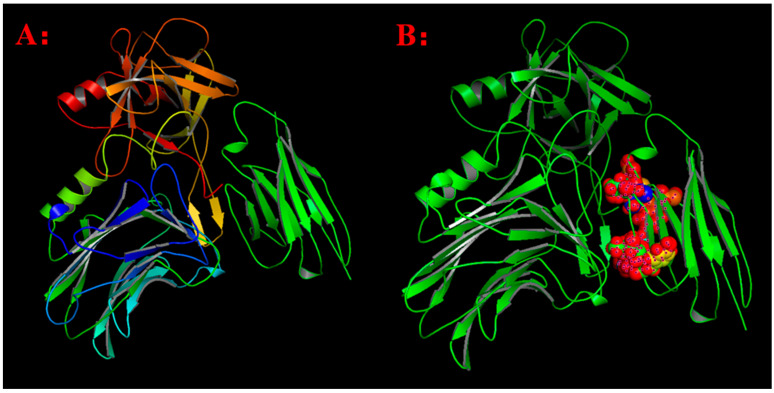
(**A**) The 3-D complex structure of antibody HM and antigen BoNT/A H chain. The green ribbon denoted the 3-D modeling structure of HM. The left ribbon structure denoted the 3-D structure of BoNT/A H chain. (**B**) The ball denoted the key amino acid residues of HM (Thr^37^/Glu^50^/Leu^54^/Ser^61^/Thr^65^/Tyr^66^/Glu^106^/Trp^108^).

**Figure 2:**
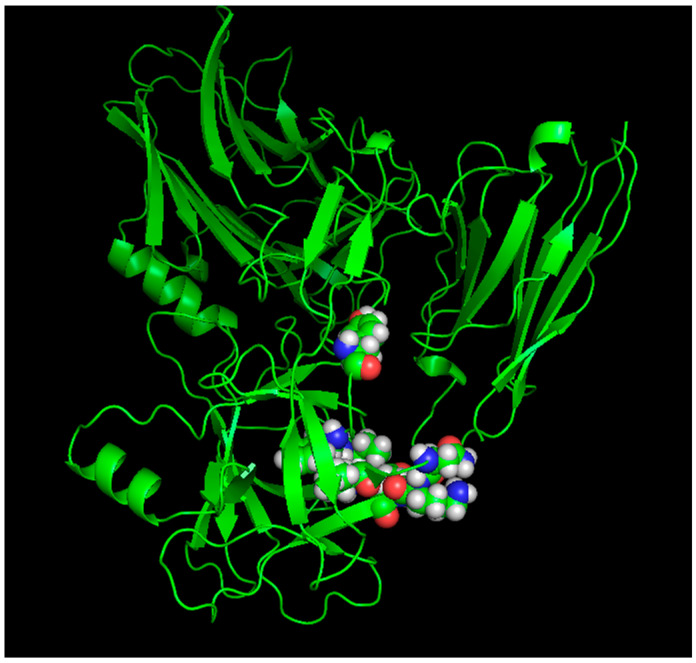
The balls denoted the key residues of BoNTA epitopes including Tyr^1155^, Phe^1160^, Ile^1161^, Val^1184^, Asn^1188^, Lys^1189^, Glu^1190^.

To investigate the specific binding mechanism and identify key binding epitope residues, the three-dimensional complex structure of HM and BoNT/A was modeled and optimized, and its stable conformation is shown in Figure 1A. Based on distance geometry and molecular graphics analysis, aromatic residues in the HM segment are concentrated around the edges of the grooves and the central pocket. Eight key residues in HM, namely Thr37H, Glu^50H^, Leu^54H^, Ser^61H^, Thr^65H^, Tyr^66H^, Glu^106H^, and Trp^108H^ (as shown in Figure 1B), form a planar binding surface and bind to the BoNT/A epitope. The binding sites of BoNT/A and HM exhibit spatial complementarity on the surface, with the central pocket serving as the anchor point, making the interaction between key epitopes of BoNT/A clearly apparent. Based on the theory of intermolecular hydrogen bonding formation and distance geometry method, the binding domain between BoNT/A and HM was determined. The antibody HM heavy chain CDR3 plays a crucial role in stabilizing BoNT/A-HM complexes by forming intermolecular hydrogen bonds. The key epitopes Tyr^1155^, Phe^1160^, Ile^1161^, Val^1184^, Asn^1188^, Lys^1189^, and Glu^1190^ in BoNT/A (as shown in Figure 2) participated in the forming van der Waals interaction and polar contacts as well as intermolecular hydrogen bonds.

### 2.2. Design of Fc Mutants Based on Virtual Screening of Fc Mutation Library

**Figure 3:**
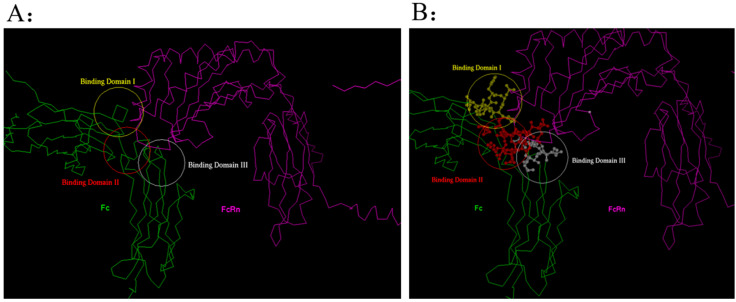
Distribution characteristics of structural domains recognized by the interaction between human Fc and FcRn. (**A**) Distribution of Fc involved in binding to FcRn regions; (**B**) Distribution of key amino acids involved in Fc binding to FcRn.

**Figure 4:**
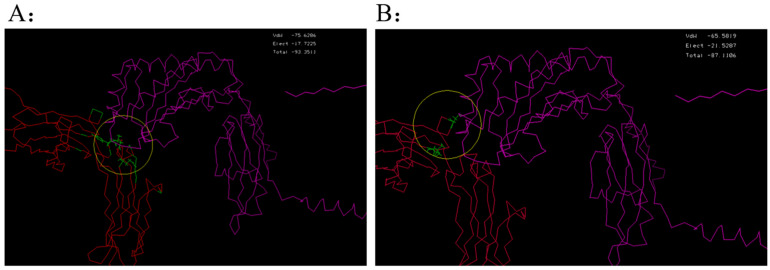
Spatial structural information of the human Fc mutants Fc5 (**A**), Fc6 (**B**), and FcRn complex.

**Figure 5:**
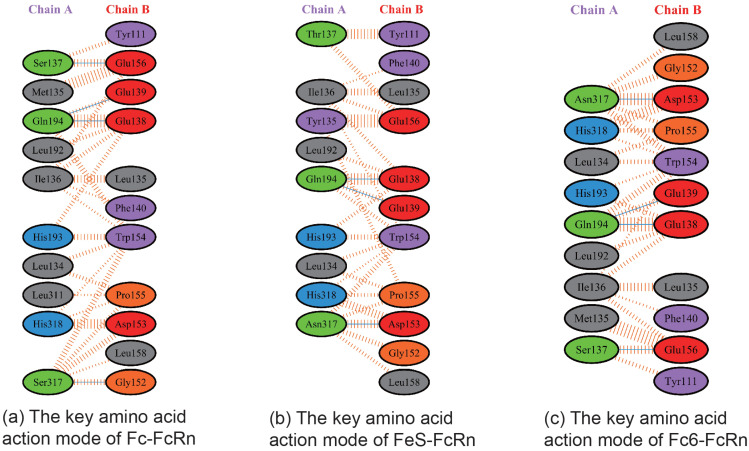
Based on the theoretical spatial conformation of Fc, Fc5, Fc6, and FcRn complexes, the key amino acid interaction modes between Fc and its mutant (Chain A) and FcRn (Chain B) were analyzed by PDBsum 1 software. The dotted orange dashed line represents Van der Waals interaction, the blue straight line represents intermolecular hydrogen bonding interaction, and the orange dashed line represents polarity interaction (the length of the dashed line represents the ability to interact, and the longer length of the dashed line represents stronger ability to interact).

By utilizing the physicochemical properties and structural parameters of amino acids, the interaction characteristics between human Fc and FcRn were investigated. Through computer virtual screening and targeted design, two potential mutation patterns were determined to have good binding activity, namely Fc5 (domain 2, Binding domain II in Figure 3) and Fc6 (domain 3, Binding domain III in Figure 3). Further comparative analysis was conducted on the binding modes (Figure 4 and Figure 5, Table 1) and interaction energies of Fc5, Fc6, and FcRn obtained under the CVFF force field (Table 2). From the intermolecular interaction modes listed in Table 1 (contact area, number of intermolecular interaction residues, and formation mode), it can be seen that Fc5 and Fc6 have better contact ability than Fc, and their interaction with FcRn is enhanced. From the interaction energy (Table 2), it can be seen that compared to wild-type human Fc, the interaction between Fc5, Fc6, and FcRn is significantly increased. Meanwhile, from Figure 4 and Figure 5, it can be seen that the structural pattern of FcRn recognized by the mutated Fc5 and Fc6 is basically consistent with that of the wild-type Fc, and the interaction epitopes have not shifted.

### 2.3. Expression, Identification and Stability of HM-Fc Mutants and BoNT/AHC Mutants

**Figure 6:**
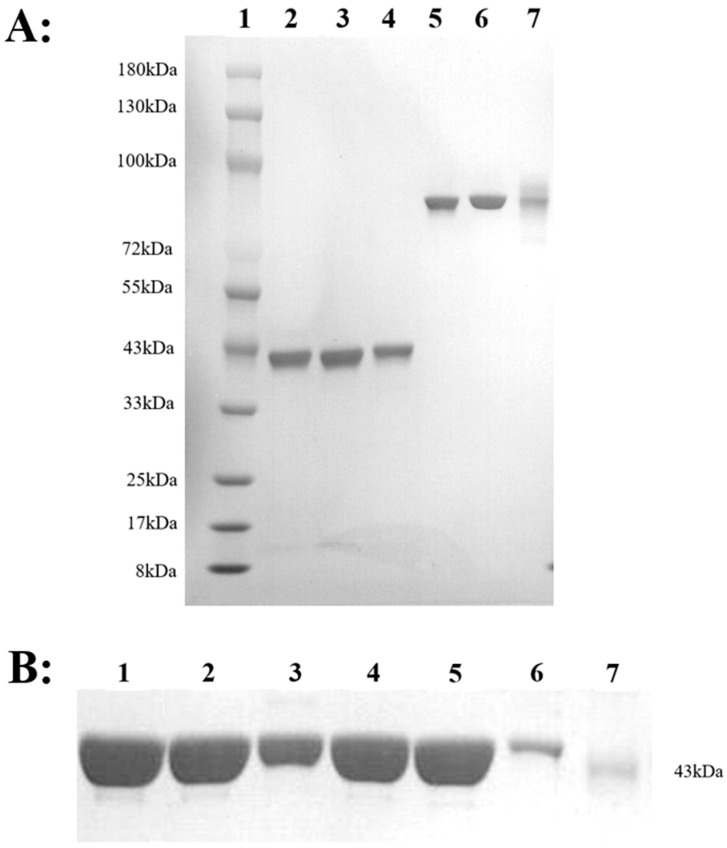
Comassie stained SDS-PAGE gels of nonreduced (NR) and reduced (R) samples of isolated antibody and antigen. (**A**) The SDS-PAGE electropherograms of HM, HM-Fc5 and HM-Fc6. Lane 1: Albumen Marker; Lane 2: HM reduced electrophoresis bands; Lane 3: HM-Fc5 reduced electrophoresis band; Lane 4: HM-Fc6 reduced electrophoresis band; lane 5: HM non-reducing electrophoresis band; Lane 6: HM-Fc5 non-reducing electrophoresis band; Lane 7: HM-Fc6 non-reducing electrophoresis bands. (**B**) The reduced electrophoresis bands of AHC and its mutants. Lane 1: AHC; Lane 2: AHC1, Lane 3: AHC2, Lane 4: AHC3, Lane 5: AHC4, Lane 6: AHC5, Lane 7: Albumen Marker.

Three purified antibodies, HM, HM-Fc5, and HM-Fc6, were obtained after expression and protein purification in the CHO-S eukaryotic system. According to the SDS-PAGE results, as shown in Figure 6A, the molecular weight of the three antibodies was approximately 80 kDa. Figure 6B showed that the molecular weight of AHC and its mutants was approximately 43 kDa. The mutation sites of the toxin are shown in Table 3 with BoNT/AHC mutation sites. In addition, we conducted preliminary stability studies of the nanobodies, in which the SEC-HPLC detection of the nanobodies were analyzed under high temperature, repeated freeze-thawing, and different pH conditions. The results showed that the three nanobodies had good stability at 4 °C, and that the purity by SEC-HPLC (280 nm) did not change significantly for at least 4 weeks. Under three repeated freeze-thaw cycles and acidic pH (3.5) conditions, the antibodies also showed good stability. However, under the condition of storing at 40 °C, the data of the SEC-HPLC (280 nM) results showed slight decreases with longer storage time when compared to a storage time of 4 weeks (Table 4), which confirmed that Fc mutations did not affect nanobody stability and provided available reference for antibody preservation.

### 2.4. Characterization of Key Epitopes in BoNT/A Recognized by HMs or SV2C

**Figure 7:**
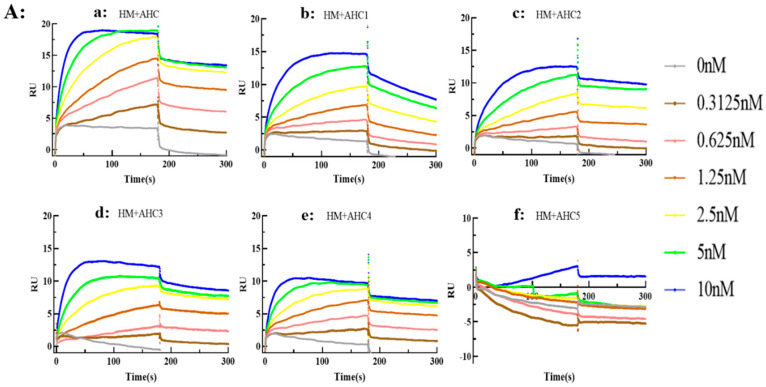

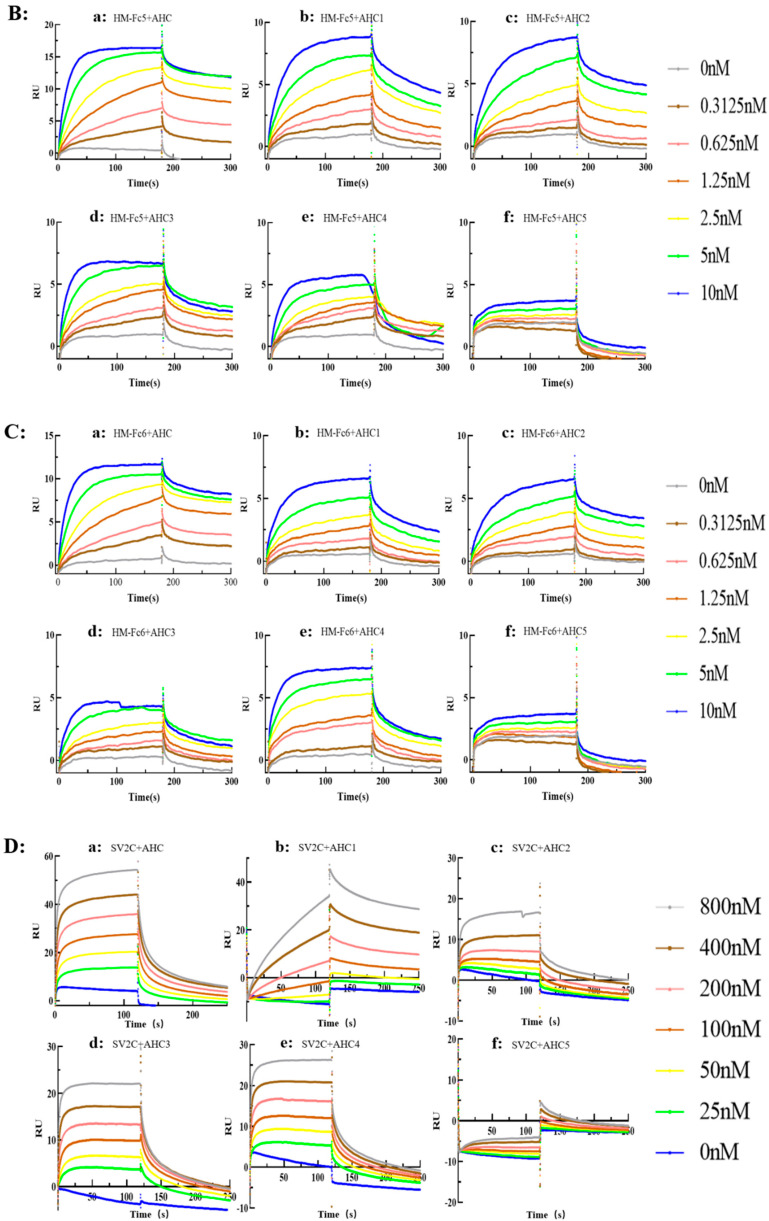
Kinetic analysis of the binding of antibodies or SV2C with a series of BoNT/AHC mutants by SPR method. (**A**) Binding curves of HM with BoNT/AHC and mutants; (**B**) Binding curves of HM-Fc5 with BoNT/AHC and mutants; (**C**) Binding curves of HM-Fc6 with BoNT/AHC and mutants; (**D**) Binding curves of SV2C with BoNT/AHC and mutants. The binding trend of the three antibodies with the BoNT/AHC mutant was the same, and the binding capacity of all antibodies with the whole mutant was decreased. The kinetic results of the binding of SV2C with the BoNT/AHC mutant also showed a decrease in the binding of the whole mutant.

Through the theoretical analysis of the interaction between HM and BoNT/A, the key sites of HM interaction with BoNT/A were obtained, and a series of mutation sites of BoNT/A were designed. The mutant fusion His-tag was expressed by the *E. coli* expression system and purified by a nickel affinity column. The mutants obtained above are named BoNT/AHC1 (single-point mutation), BoNT/AHC2 (two-point mutation), BoNT/AHC3 (single-point mutation), BoNT/AHC4 (three-point mutation), and BoNT/AHC5 (comprehensive mutants 1–4, complete mutants). The antibodies HM, HM-Fc5, and HM-Fc6 were exposed to the mutants and their binding affinity was assayed using the BiAcore method to detect the kinetics of HMs and BoNT/A or BoNT/A mutants. After three repeated experiments, the results are as follows: As shown in Figure 7A–C, the binding of HM with BoNT/AHC1~4 showed few or no reduction, while AHC5, which contained all predicted residues replaced by alanine lost the binding capacity. HM-Fc5 and HM-Fc6 exhibited the same binding function as HM. Table 5 was obtained by collating Biacore data to directly show the binding, dissociation, and affinity of the antibodies (HM, HM-Fc5, and HM-Fc6) to the AHC mutants, corresponding to the trend of the binding curve, and comparing the KD values of the mutants to the KD values of wild-type BoNT/A AHC.

The kinetics of SV2C (cell surface receptor of botulinum) and BoNT/A AHC or AHC mutants were also tested. As shown in Figure 7D, similar to the results of the antibody-AHCs kinetic data, only AHC5 completely lost the binding capacity with the receptor SV2C, suggesting that the key residues of AHC recognized by the three antibodies or SV2C seemed overlapped, which included Tyr^1155^, Phe^1160^, Ile^1161^, Val^1184^, Asn^1188^, Lys^1189^, and Glu^1190^, at least.

### 2.5. Biological Effect of Antibodies to Antagonize the Toxin Receptor SV2C

**Figure 8:**
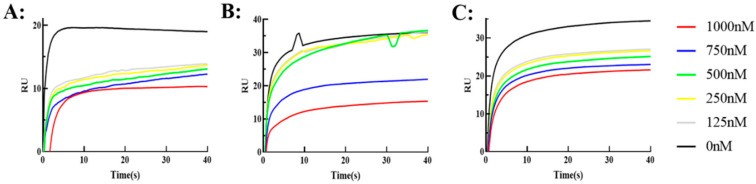
Antibodies ((**A**): HM, (**B**): HM-Fc5, and (**C**): HM-Fc6) competed with SV2C to bind with AHC, respectively. HMs were incubated with different concentrations of AHC and then analyzed by Biacore to draw their binding curves with SV2C.

The biological effects of the three antibodies were evaluated by a BiAcore-based binding assay, the toxin receptor SV2C was conjugated on a CM5 chip, and the BoNT/AHC was incubated with different concentrations of the three antibodies at room temperature for 30 min, and then the binding kinetics were analyzed. The results are shown in Figure 8, showing that the ability of the antibody to competitively bind with BoNT/A is enhanced as the antibody concentration increases, and the ability of BoNT/A to bind with SV2C is completely inhibited by the three antibodies at high concentrations. The results suggest that the key epitopes of the antibody overlap with those of SV2C, so the possible mechanism of its potential in vivo protective activity may be to block the binding of intracellular toxins, and Fc modification does not affect the blocking activity of HM.

### 2.6. In Vivo Protection Activity of the Antibodies in Human FcRn Transgenic Mice

**Figure 9:**
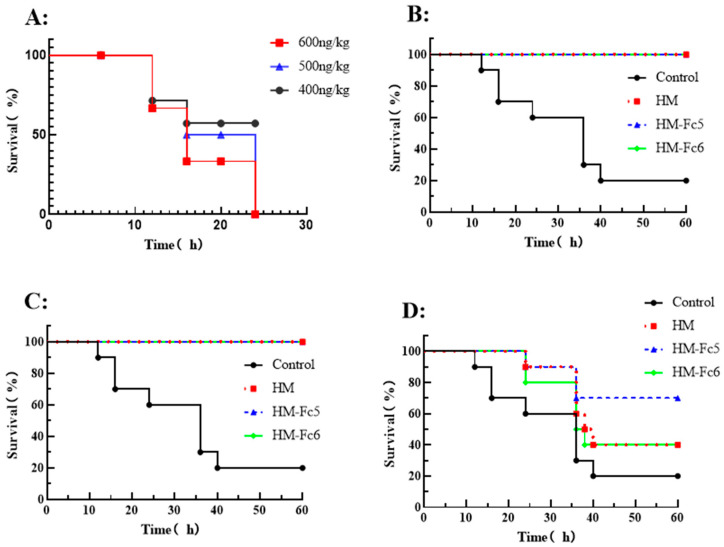
Neutralizing effect of antibodies in poisoned human FcRn transgenic mice model. (**A**): The survival curve of the BoNT/A challenge in transgenic mice (n = 5); (**B**–**D**): The protective survival curve of the three antibodies in LD_100_ of BoNT/A challenged mice. The antibody doses were 500 μg kg^−1^ (**B**), 5 μg kg^−1^ (**C**), and 0.5 μg kg^−1^ (**D**), respectively (n = 10).

To further verify the neutralizing effect of the antibodies against the toxin, 16~18 g human FcRn transgenic mice (8-week-old) were selected for the BoNT/A challenge dose test. The BoNT/A toxin stored in the laboratory was diluted to a series of concentrations, and each mouse was intraperitoneally injected with 100 μL toxin. The survival status and mortality of the mice was then observed. The survival curve of the mice is shown in Figure 9A. In the 500 ng kg^−1^ toxin group, the survival rate of mice was 0%, and in the 400 ng kg^−1^ group, it was over 50%, suggesting that the dose of 500 ng kg^−1^ BoNT/A could be set as the lethal dose (LD_100_) of BoNT/A in human FcRn transgenic mice models.

Neutralizing activity of antibodies (HM, HM-Fc5, and HM-Fc6) was verified by BoNT/A challenge assay in human FcRn transgenic mice. A range of antibody concentrations (500 μg kg^−1^, 5 μg kg^−1^ and 0.5 μg kg^−1^) were incubated with LD_100_ of BoNT/A in a volume of 200 μL for 30 min at room temperature and then injected intraperitoneally into mice. The PBS+BoNT/A group was set as the control to observe the survival of poisoned mice. As shown in Figure 9B,C, all mice in the 500 μg kg^−1^ and 5 μg kg^−1^ groups survived, while the low concentration of 0.5 μg kg^−1^ antibody group only protected about 50% of the mice (Figure 9D), reflecting a dose-dependent protective effect of antibodies in vivo. However, although a low dose of antibodies did not fully protect the mice, they successfully prolonged the survival time of mice. Meanwhile, the three antibodies showed similar protection effect, indicating that the antibody modified by Fc5 or Fc6 mutation does not affect the in vivo activity of neutralizing the toxin.

### 2.7. Fc-Modified Antibodies Had Prolonged In Vivo Half-Life in Post-Injection Neutralizing Assay

**Figure 10:**
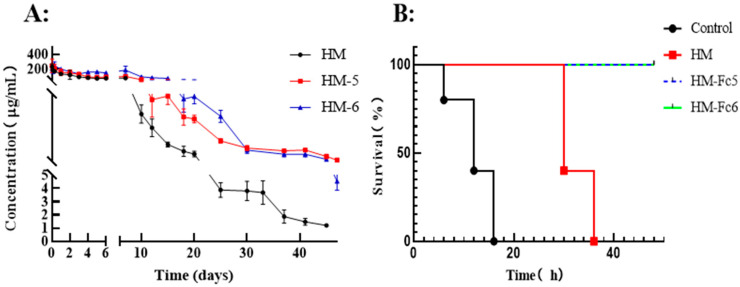
Plot of pharmacokinetic (**A**) and pharmacodynamic (**B**) results of antibody HMs in human FcRn transgenic mice. A: The curve of antibody concentration over time in transgenic mice, with initial injection of 500 μg piece^−1^ (n = 5). B: on the 50th day post-injection of antibody, 10 times of the lethal dose of the toxin (4 μg kg^−1^) was injected into hFCRN mice, and the survival curve of each group of mice differed between the three antibody groups (N = 5).

In human FcRn transgenic mice, we determined the in vivo pharmacokinetic and pharmacodynamic characters of the antibodies. Mice were injected with 500 μg antibodies on Day 1, respectively, and the antibody concentration in serum was measured at different time points by sandwich ELISA. As shown in Figure 10A, HM-Fc5 and HM-Fc6 have a longer half-life than the maternal antibody HM. On the 50th day post-injection, 10 times of the lethal dose of BoNT/A was intraperitoneally injected into mice. As shown in Figure 10B, both HM-Fc5 and HM-Fc6 were effective in protecting mice from death, while all mice in the HM group died within 36 h, and in the control group without antibody administration all died within 16 h. The results showed that HM-Fc5 and HM-Fc6 could effectively prolong the retention time in vivo under the same initial conditions and thereby reduce medication intervals.

## 3. Discussion

BoNT/A (botulinum toxin A) is a neurotoxin produced by *Clostridium botulinum*. It is one of the strongest known neurotoxins and can block the release of acetylcholine by cleaving SNAP-25, thereby blocking nerve signaling and causing muscle paralysis [27]. The interaction between antibodies and antigens is the core mechanism for the immune system to recognize and eliminate foreign pathogens. Antibodies bind to antigens through their specific binding sites, forming antigen antibody complexes that can trigger a series of immune responses such as neutralization, regulation of phagocytosis, and complementary activation [28]. With the significant increase in antibody antigen eutectic structures in the protein database (PDB), large-scale analysis can now be performed to predict epitope features and reveal immune molecule recognition. Rui Jin Ji et al.’s study explored a new strategy for the treatment of acute myeloid leukemia (AML). The research team utilized base editors (BEs) and lead editors (PE) to precisely edit the CD123 antigen epitopes in hematopoietic stem cells and progenitor cells (HSPCs) to protect healthy cells from cytotoxicity caused by CAR-T cell immunotherapy, demonstrating the potential of precise epitope editing technology in immunotherapy for malignant diseases [29]. In addition, the availability of natural antigen structures provides conditions for studying the conformational changes of antigens caused by antibody binding. Research has shown that precise localization of epitopes is crucial for the development and optimization of biomedical applications such as vaccines, diagnostic kits, and immunotherapy.

In the development of antibody drugs, the modification of Fc fragments is an important research direction aimed at improving the efficacy and stability of antibodies. Single domain antibodies (VHH or nanobodies) have become a research hotspot in recent years due to their small molecular weight and high stability [30]. However, nanobodies typically have shorter half-life, which may limit their widespread use in clinical applications. In our study, a nanobody HM was fused with Fc or Fc mutants, which might enhance their affinity to bind with FcRn receptor. Then, three nanobodies were analyzed with their in vivo pharmacokinetic character in human FCRN (2) transgenic mice. The results showed that the half-life of two mutated nanobodies was longer than that of parent HM. When an initial dose of 25 mg kg^−1^ was given, HM was undetectable on day 45 (lower than 1 μg mL^−1^), while at the same time point, the concentrations of Fc 5 and Fc 6 could be detected at 10 μg mL^−1^. Similar to the reported Ig-Fc mutants, e.g., YTE and other mutations, our Fc mutants could effectively prolong the half-life of nanobodies, which will not only improve the efficacy but also reduce the frequency of administration to improve patient compliance and quality of life.

In this study, we also conducted structural and functional studies on the interaction between nanostructured HM and BoNT/A, identifying key epitopes in BoNT/A, which were identified by its parent functional antibody HM and two modified antibodies HM-Fc5 and HM-Fc6. In detail, we constructed the 3-D complex structure of HM and antigen theoretically and predicted rationally the key epitopes in the antigen identified by HM, as Tyr^1155^, Phe^1160^, Ile^1161^, Val^1184^, Asn^1188^, Lys^1189^, and Glu^1190^. The following experimental results showed that the predicted epitopes were correct, and these sites were also important to bind the membrane receptor SV2C, which could help toxin to enter cells. Furthermore, Fc-revised antibodies could help prolong their own half-life in human FcRn-transgenic mice, possibly due to stronger efficiency of FcRn-induced IgG recycling. However, Fc mutations may affect the stability of antibody results, so we validated the stability of three nanobodies under different conditions. The results showed that long-term storage at 4 °C, repeated freeze-thawing, or lower pH had little effect on the stability of the antibodies, while at 40 °C for 4 weeks, the stability decreased slightly, indicating that Fc mutations did not affect the stability of the nanobodies. In all, we prepared two revised anti-BoNT/A neutralizing nanobodies with overlapped epitope with toxin receptor SV2C, which possessed longer half-life, lower dosing frequency, and, most importantly, stronger prevention effect as a potential antagonist in vivo for future application.

## 4. Methods and Materials

### 4.1. Nano-Antibody HM Structural Modeling

The amino-acid residue sequences of an anti-BoNT/AHC nanobody, named as HM, were compared with the primary sequences of all VHHs deposited in the Protein Data Bank using BLAST program (1.4.0). The Complementary Determinant Region (CDR) of HM variable domain was defined using Kabat method [31]. The best match template for HM was the protein with PDB code of 5L21, which has 95% identity of residue sequences. Consequently, the framework of HM was superimposed on the corresponding framework, using a rigid-body superimposition program. Based on Homology modeling methods deposited in InsightII 2000 program (Molecular Simulations, San Diego, CA, USA), the 3-D spatial structure of HM was modeled. To reduce steric hindrance conflicts and optimize bond length and angle, after fixing the position of the alpha carbon atom in the FR region, the Discover module of InsightII 2000 program was used to perform 10,000 steps of energy minimization on the CDR model structure.

### 4.2. Nano-Antibody HM and BoNT/AHC Complex Structure Constructing

Based on the crystal structure of BoNT/AHC, using Docking methods (Molecular Simulations, San Diego, CA, USA), the 3-D complex structure of HM and BoNT/AHC was modeled to show the ‘key’ residues and their interactions in the interface. To reduce steric hindrance and optimize non bonding distance, bond angle, and hydrogen bonding, the interaction region between BoNT/AHC and HM was minimized in 10,000 steps after fixing the remaining structural positions. After minimizing the energy, the interaction between HM and BoNT/A was analyzed.

### 4.3. Mutant Design and Preparation of BoNT/AHC

The examination of the molecular surface where HM binds to the BoNT/AHC receptor binding domain indicates that the pocket at the bottom of the center can provide an anchor point for the epitope. Referring to the research of Wei et al. [14], a three-dimensional optimized composite structure based on BoNT/AHC-HM was used to predict the binding sites between the two, and a series of BoNT/AHC mutants were designed.

This study used the gold standard method of alanine scanning mutagenesis for epitope validation [32], aiming to accurately identify key antigenic epitope residues in the BoNT/A Hc domain. Firstly, the DNA sequence encoding the BoNT/AHC mutant was obtained through polymerase chain reaction (PCR). BoNT/AHC mutants included BoNT/AHC1 (Tyr 1155 ALA), BoNT/AHC2 (Phe 1160 ALA, Ile 1161 ALA), BoNT/AHC3 (Val 1184 ALA), BoNT/AHC4 (Asn 1188 ALA, Lys 1189 ALA, Glu 1190 ALA), BoNT/AHC5 (Tyr 1155 ALA, Phe 1160 ALA, Ile 1161 ALA, Val 1184 ALA, Asn 1188 ALA, Lys 1189 ALA, Glu 1190 ALA). These mutants were generated using overlapped PCR methods to introduce the GCT (coding alanine) into the mutation site. All the plasmids constructed above were confirmed by DNA sequencing. The BoNT/AHC or mutants were fused with His6 tag and expressed in *E. coli* BL21 and purified with nickel-chelated affinity column by standard methods from the supernatant of cell lysate. Then the purified proteins were analyzed by sodium dodecyl sulfate-polyacrylamide gel electrophoresis (SDS-PAGE).

### 4.4. Design, Expression and Purification of HM, HM-Fc5 and HM-Fc6

Previously, we utilized the crystal structure of the complex formed by the interaction between human Fc and FcRn (PDB registration number: 4WI2), selected appropriate molecular force field CVFF parameters, and performed hydrogen atom coordinate assignment and molecular mechanics optimization on the theoretical spatial structure of the complex formed by the interaction between human Fc and FcRn using IBM graphics workstation and molecular simulation software package InsightII 2005. The convergence criterion of the steepest descent method was 0.05 kCal mol^−1^, and the optimization step size was 20,000 steps; Conjugate gradient convergence criterion 0.01 kCal mol^−1^, optimized step size 50,000 steps). On the basis of obtaining a stable spatial conformation of the complex, important amino acid residues involved in the binding of human Fc to FcRn were theoretically determined using distance geometry, intermolecular hydrogen bonding, and computer graphics techniques.

By examining the physicochemical properties of amino acids and utilizing their interaction energy changes, important amino acids were theoretically subjected to site directed mutagenesis and interaction energy calculations. Subsequently, potential affinity enhancing mutants (named Fc5 and Fc6, respectively) were combined and designed. This design introduces two enzyme cleavage sites, NotI and PmeI, at both ends of the target gene. These genes were synthesized by Azenta Life Science, and the mutated Fc region was cloned into the HM antibody expression vector.

Thaw ExpiCHO-S™ cells into a 125 mL shake flask containing pre-warmed ExpiCHO™ expression medium in a 37 °C, 8% CO_2_ incubator until cell density reaches 4 × 10^6^~6 × 10^6^ mL^−1^. Dilute the cells to 0.2 × 10^6^~0.3 × 10^6^ mL^−1^ and continue incubating.

Adjust the cell density to 3 × 10^6^ the day before transfection and 6 × 10^6^ mL^−1^ on the day of transfection. Plasmid DNA (100 μg 100^−1^ mL^−1^) and ExpiFectamine™ CHO Transfection Reagent (320 μL) were diluted in low-temperature OptiPRO™ SFM Medium. After a 5-min mixed incubation at room temperature, slowly added the complex to the cell suspension and mix gently. 18–22 h post-transfection, ExpiFectamine™ CHO Enhancer (600 μL) and ExpiCHO™ Feed (24 mL) were added. After 8 days of culture, antibody proteins were harvested from the culture supernatant, initially purified using the HiTrap MabSelet Protein A Column, eluted with 150 nM acetic acid on the Akta Purification System, and stored in PBS (pH = 7.4). The concentration of antibody was determined by the BSA concentration determination method, while the preliminary identification of antibodies is accomplished by SDS electrophoresis.

### 4.5. Purification of Expressed Antibodies

Purification of antibodies using AKTA prime plus purifier and HiTrap MabDelete protein A column. According to the program’s parameter setting process: After turning on the purifier, set the flow rate to 1 mL min^−1^, and first rinse the pipeline with sterilized injection water for 20 min; Replace the balance buffer Wash 2 (1 L: K_2_HPO_4_-20.54 g, KH_2_PO_4_-55.8 g) and rinse for 20 min. Subsequently, the flow rate was reduced to 0.5 mL min^−1^ and the purification column was loaded to avoid adverse effects on the column caused by excessive flow rate. Raise the flow rate to 1 mL min^−1^, rinse the column with Wash 2 first, and load the sample when the UV absorption AU value drops to baseline. After loading is complete, rinse again with Wash 2. When the AU value drops to baseline, wash the target protein with 0.15 M acetic acid. When the AU value increases, collect the target protein flowing out of the sample port into a clean 15 mL centrifuge tube, and immediately adjust the pH of the solution to neutral using 1 M Tris HCl (pH = 9) in a super clean bench. Then transfer it to a 30 kDa ultrafiltration tube and add 10 mL of 1 × PBS ultrafiltration displacement buffer. Centrifuge at 4 °C, 5000 r, for 30 min, and repeat 3 times. After purification and collection, rinse the column with Wash 2 and regenerate it with 0.1 M NaOH for 10 min. Rinse the pipeline and column again with Wash 2, and finally store the column in 20% alcohol.

The purified protein was determined by BSA concentration assay and preliminarily identified by SDS electrophoresis.

### 4.6. SPR Assays

Immobilized HM, HM-Fc5 or HM-Fc6 on the surface of the Biacore T200 sensor chip (Protein A) was set as fixation, different concentrations of antigen AHC or AHC mutants were used as mobile phases, and the reaction curves were recorded through the sensor chip, and repeat the experiment three times. The binding constant (Ka) and dissociation constant (Kd) at each concentration were then calculated using the 1:1 Langmuir binding model, and the equilibrium constant (KD = Kd Ka^−1^) was calculated to reflect the affinity between the antigen and the antibody.

### 4.7. In Vivo Protection Activity of Antibodies Against BoNT/AHC Toxin in Transgenic Mouse Model

16–18 g C57BL/6Smok-Fcgrt^em2 (hFCGRT) Smoc^ mice [33] (hFCRN (2) mice). Cat.No. NM-HU-190070), purchased from Shanghai Model Biology Center of Co., Ltd. (Shanghai, China), and randomly divided into groups for unified feeding in a standardized experimental animal room. The BoNT/A toxin preserved in our laboratory was diluted serially to a volume of 100 μL, and each mouse was administered by intraperitoneal injection. The survival status and mortality of the mice were observed. The lethal dose (LD_100_) of the BoNT/A in in vivo challenge model was confirmed.

Then, three antibodies, HM, HM-Fc5 and HM-Fc6, were used to protect the mice injected with the toxin. 500 μg kg^−1^ of antibody was incubated with LD_100_ of BoNT/A in a total volume of 200 μL, incubated at room temperature for 30 min, then the mice were injected intraperitoneally with the mixture. BoNT/A diluted with PBS was set as the control group [26].

The neutralizing activity of the purified antibody was also identified using transgenic mice (hFCRN (2)). Mice (5 per group) were intraperitoneally injected with 500 μg of antibody. After 50 days, based on the results of the BoNT/A attack dose test, mice were inoculated with 10 times the lethal dose (LD) of BoNT/A toxin, and the same batch of purchased mice (without antibody injection) were used as the control group to observe their survival status.

All data were analyzed and plotted using GraphPad Prism 8.

The experiment used isoflurane (VetOne, headquartered in Denver, CO, USA) to anesthetize mice through a calibrated evaporator. Induction was initiated with 4% isoflurane in 100% oxygen at a flow rate of 1.5 L/min in an acrylic chamber, and maintained with 1.5–2% isoflurane after loss of righting reflex. At the end of the experiment, the carbon dioxide suffocation method was used: the mice were placed in a clean container and carbon dioxide was slowly introduced. As the concentration of carbon dioxide increases, animals will slowly die without pain. Check the condition of the mice for 10 min and examine them one by one to ensure complete death.

## Figures and Tables

**Table 1:** Comparison of the interaction energies between Fc, mutants (i.e., Fc5, Fc6) and FcRn complexes (kCal/mol).

Interacting Molecules	Interaction Energy		
	van der Waals Energy	Electrostatic Energy	Total Energy
Fc-FcRn	−67.18	−17.17	−84.35
Fc5-FcRn	−75.63	−17.72	−93.35
Fc6-FcRn	−65.58	−21.53	−87.11

**Table 2:** Comparison of amino acid residues and interaction area between Fc mutants (Fc5, Fc6) and FcRn, as well as the number of hydrogen bonds and non-bonding interactions (* represents: the ahead value of the ratio denoted Fc, while the back denoted FcRn).

Interacting Molecules	Boundary Area of Action (A^2^) *	Interface Functional Residues *	Number of Hydrogen Bonds	Number of Non Key Interactions
Fc-FcRn	670:643	9:11	3	75
Fc5-FcRn	670:643	9:11	3	75
Fc6-FcRn	618:618	10:11	4	65

**Table 3:** Amino acid sequences of AHC and mutants (red represents mutations).

Name	Amino Acid Sequence (1141 to 1200)
AHC	GSVMTTNIYL NSSLYRGTKF IIKKYASGNK DNIVRNNDRV YINVVVKNKE YRLATNASQA
AHC1	GSVMTTNIYL NSSLARGTKF IIKKYASGNK DNIVRNNDRV YINVVVKNKE YRLATNASQA
AHC2	GSVMTTNIYL NSSLYRGTKA AIKKYASGNK DNIVRNNDRV YINVVVKNKE YRLATNASQA
AHC3	GSVMTTNIYL NSSLYRGTKF IIKKYASGNK DNIVRNNDRV YINAVVKNKE YRLATNASQA
AHC4	GSVMTTNIYL NSSLYRGTKF IIKKYASGNK DNIVRNNDRV YINVVVKAAA YRLATNASQA
AHC5	GSVMTTNIYL NSSLARGTKA AIKKYASGNK DNIVRNNDRV YINAVVKAAA YRLATNASQA

**Table 4:** SEC-HPLC stability test data of HMs under different conditions (280 nm).

	Time/Frequency	HM	HM-5	HM-6
4 °C	0 h	99.3%	99.2%	94.9%
	24 h	99.1%	99.2%	93.8%
	2 W	99.3%	99.2%	95.7%
	4 W	99.3%	99.2%	94.5%
40 °C	2 W	99.4%	99.2%	95.4%
	4 W	98.8%	98.5%	94.0%
Freeze-thaw (−80 °C)	1rd	99.1%	99.2%	94.4%
	2rd	99.1%	99.2%	94.3%
	3rd	99.0%	99.2%	94.3%
pH 3.5 (4 °C)	0 h	99.5%	99.5%	99.4%
	4 h	99.4%	99.4%	99.3%
	24 h	95.1%	95.2%	95.2%

**Table 5:** Association (ka) and dissociation (kd) rate constants and equilibrium dissociation constant (KD) of HMs or SV2C binding with BoNT/AHC mutants.

	Name		AHC	AHC1	AHC2	AHC3	AHC4	AHC5
HM	ka	(1/Ms)	(7.12 ± 0.02) × 10^6^	(2.91 ± 0.01) × 10^6^	(3.86 ± 0.01) × 10^6^	(5.67 ± 0.05) × 10^6^	(6.36 ± 0.03) × 10^6^	(6.41 ± 0.02) × 10^3^
	Kd	(1/s)	(7.28 ± 0.01) × 10^−5^	(2.35 ± 0.01) × 10^−3^	(2.16 ± 0.02) × 10^−4^	(1.39 ± 0.02) × 10^−4^	(2.65 ± 0.03) × 10^−4^	(1.27 ± 0.02) × 10^−3^
	KD	(M)	(1.022 ± 0.007) × 10^−11^	(8.076 ± 0.035) × 10^−10^	(5.596 ± 0.055) × 10^−11^	(2.451 ± 0.044) × 10^−11^	(4.167 ± 0.053) × 10^−11^	(1.981 ± 0.032) × 10^−7^
HM-Fc 5	ka	(1/Ms)	(1.022 ± 0.007) × 10^−11^	(2.72 ± 0.01) × 10^6^	(3.20 ± 0.02) × 10^6^	(7.27 ± 0.04) × 10^6^	(6.53 ± 0.03) × 10^6^	(1.08 ± 0.01) × 10^4^
	kd	(1/s)	(9.60 ± 0.05) × 10^−5^	(2.86 ± 0.01) × 10^−3^	(1.09 ± 0.01) × 10^−3^	(4.61 ± 0.03) × 10^−4^	(8.01 ± 0.04) × 10^−4^	(2.03 ± 0.02) × 10^−3^
	KD	(M)	(1.943 ± 0.015) × 10^−11^	(1.052 ± 0.006) × 10^−9^	(3.406 ± 0.041) × 10^−10^	(6.341 ± 0.05) × 10^−11^	(1.227 ± 0.012) × 10^−10^	(1.880 ± 0.025) × 10^−7^
HM-Fc 6	Ka	(1/Ms)	(5.96 ± 0.03) × 10^6^	(1.69 ± 0.01) × 10^6^	(1.94 ± 0.01) × 10^6^	(8.64 ± 0.04) × 10^6^	(1.80 ± 0.01) × 10^6^	(4.31 ± 0.02) × 10^3^
	kd	(1/s)	(4.08 ± 0.02) × 10^−4^	(3.65 ± 0.02) × 10^−3^	(1.15 ± 0.01) × 10^−3^	(1.06 ± 0.01) × 10^−3^	(1.09 ± 0.01) × 10^−3^	(5.08 ± 0.05) × 10^−6^
	KD	(M)	(6.846 ± 0.045) × 10^−11^	(2.160 ± 0.018) × 10^−9^	(5.928 ± 0.062) × 10^−10^	(1.227 ± 0.015) × 10^−10^	(6.056 ± 0.061) × 10^−10^	(1.179 ± 0.015) × 10^−9^
SV2C	ka	(1/Ms)	(2.58 ± 0.03) × 10^5^	(2.11 ± 0.02) × 10^4^	(2.68 ± 0.03) × 10^4^	(6.78 ± 0.07) × 10^5^	(1.82 ± 0.02) × 10^5^	(4.00 ± 0.04) × 10^4^
	kd	(1/s)	(0.014 ± 0.001)	(7.91 ± 0.08) × 10^−4^	(0.0021 ± 0.0002)	(0.0149 ± 0.001)	(0.0054 ± 0.0003)	(0.0077 ± 0.0004)
	KD	(M)	(5.43 ± 0.43) × 10^−8^	(3.75 ± 0.30) × 10^−8^	(7.84 ± 0.78) × 10^−8^	(2.20 ± 0.18) × 10^−8^	(2.97 ± 0.21) × 10^−8^	(1.93 ± 0.15) × 10^−7^

## Data Availability

The data that support the findings of this study are available from the corresponding author upon reasonable request.
